# Complete biotransformation of cellulose to starch *in vitro*

**DOI:** 10.1093/nsr/nwaf503

**Published:** 2025-11-14

**Authors:** Jingting Wang, Yunjie Li, Mingyuan Lu, Qingqing Guo, Yuanyuan Chen, Yuan Li, Yanhong Jing, Zhenyu Zhai, Ting Shi, Yuzhen Zhang, Biwang Jack Jiang, Xiang Sheng, Yi-Heng P Job Zhang

**Affiliations:** State Key Laboratory of Engineering Biology for Low-Carbon Manufacturing, Tianjin Institute of Industrial Biotechnology, Chinese Academy of Sciences, China; In vitro Synthetic Biology Center, Tianjin Institute of Industrial Biotechnology, Chinese Academy of Sciences, China; State Key Laboratory of Engineering Biology for Low-Carbon Manufacturing, Tianjin Institute of Industrial Biotechnology, Chinese Academy of Sciences, China; In vitro Synthetic Biology Center, Tianjin Institute of Industrial Biotechnology, Chinese Academy of Sciences, China; Suzhou NanoMicro Technology Co Ltd, China; In vitro Synthetic Biology Center, Tianjin Institute of Industrial Biotechnology, Chinese Academy of Sciences, China; State Key Laboratory of Engineering Biology for Low-Carbon Manufacturing, Tianjin Institute of Industrial Biotechnology, Chinese Academy of Sciences, China; State Key Laboratory of Engineering Biology for Low-Carbon Manufacturing, Tianjin Institute of Industrial Biotechnology, Chinese Academy of Sciences, China; In vitro Synthetic Biology Center, Tianjin Institute of Industrial Biotechnology, Chinese Academy of Sciences, China; In vitro Synthetic Biology Center, Tianjin Institute of Industrial Biotechnology, Chinese Academy of Sciences, China; In vitro Synthetic Biology Center, Tianjin Institute of Industrial Biotechnology, Chinese Academy of Sciences, China; University of Chinese Academy of Sciences, China; State Key Laboratory of Engineering Biology for Low-Carbon Manufacturing, Tianjin Institute of Industrial Biotechnology, Chinese Academy of Sciences, China; In vitro Synthetic Biology Center, Tianjin Institute of Industrial Biotechnology, Chinese Academy of Sciences, China; In vitro Synthetic Biology Center, Tianjin Institute of Industrial Biotechnology, Chinese Academy of Sciences, China; Suzhou NanoMicro Technology Co Ltd, China; State Key Laboratory of Engineering Biology for Low-Carbon Manufacturing, Tianjin Institute of Industrial Biotechnology, Chinese Academy of Sciences, China; University of Chinese Academy of Sciences, China; State Key Laboratory of Engineering Biology for Low-Carbon Manufacturing, Tianjin Institute of Industrial Biotechnology, Chinese Academy of Sciences, China; In vitro Synthetic Biology Center, Tianjin Institute of Industrial Biotechnology, Chinese Academy of Sciences, China; University of Chinese Academy of Sciences, China

## Abstract

A multi-enzyme molecular machine enables the complete conversion of cellulose into starch, potentially unlocking agricultural residues and grass as a new feed and food source for non-ruminant animals and humans.

Feeding a growing global population is among humanity’s most urgent challenges [1–3]. For millennia, humans have relied on annual crops; each year we sow seeds, harvest the grains, and reserve part of them to plant again [4]. Starch-laden grains supply the bulk of both human dietary calories and the energy ration of non-ruminant livestock [2,5].

Although cellulose is the planet’s most abundant and renewable polysaccharide [5], humans and other non-ruminant animals cannot survive on lignocellulosic biomass, such as wood, grass and agricultural residues. Ruminants and termites can live on lignocellulosic biomass because symbiotic cellulolytic microorganisms in their guts can hydrolyze lignocellulose into fermentable sugars and/or ferment them into volatile fatty acids that the host can absorb. In some special cases, cellulolytic microorganisms could hydrolyze cellulose *ex vivo*, assimilate the resulting soluble sugars, and even re-assemble them into starch *in vivo*. Unfortunately, this natural bioconversion suffers from very low yields and low productivity (Fig. S1a and Table S1).

High-yield biotransformation of cellulose into starch would give humans and non-ruminant animals access to an entirely new calorie resource and could trigger a new wave of agricultural revolution [5]. Zhang *et al*. recently made the first attempt to convert cellulose to starch by combining *in vitro* biotransformation (*iv*BT) and microbial fermentation [6]. The theoretical yield of *in vitro* cellulose-to-starch (iC2S) comprising cellobiose phosphorylase (CBP) and potato α-glucan phosphorylase (PGP) was 50% because half of the glucose units were converted to ethanol under anaerobic conditions [6] or microbial protein under aerobic conditions [7]. In practice, these iC2S yields were low, approximately 14%–18% [6,7] (Table S1).

To address the low iC2S yields [6,7], we designed and validated an *in vitro* synthetic enzymatic pathway that can convert all glucose units of cellulose to those of starch, i.e. a theoretical yield of 100% (Fig. 1a). This directed rearrangement of glycosidic bonds from β-1,4- to α-1,4-glucans features several-fold enhancements in theoretical yields and volumetric productivity (Fig. 1b and c). At the initial trial, we designed a supplementary route with polyphosphate glucokinase (PPGK) and phosphoglucomutase (PGM) for the conversion of glucose to glucose 1-phosphate (G1P), which may be directed to synthesize starch (Fig. S1b and c). However, the four-enzyme cocktail (containing CBP, PPGK, PGM and PGP) cannot make more starch than the CBP/PGP cocktail (Fig. S1d). Because bioenergetic analysis suggests that adenosine diphosphate (ADP)-glucose was a more energetically favored intermediate than G1P, we redesigned the *in vitro* cellulose-to-amylose (iC2A) pathway (Fig. 1a) by the substitution of PGP with ADP-glucose pyrophosphorylase (AGP) and starch synthase (SS). All recombinant enzymes were purified to a single band (Fig. S2).

**Figure 1.**
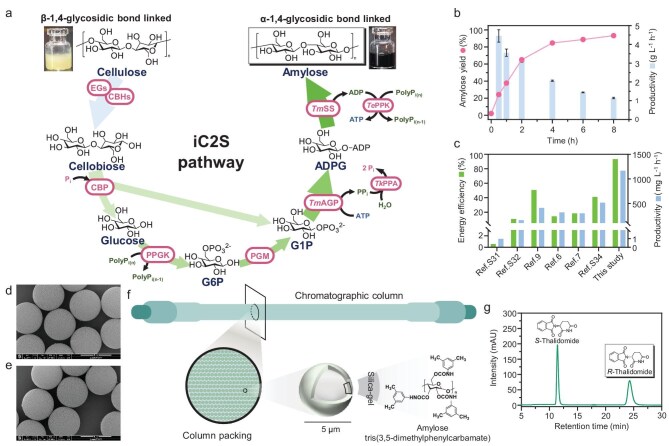
Schematic illustration of the iC2S biotransformation mediated by a multiple-enzyme molecular machine (a), where it includes endoglucanase (EG), cellobiose hydrolase (CBH), CBP, PPGK, PGM, AGP and SS, as well as two supplementary enzymes—PPK and PPA. (b) The profiles of amylose yield and volumetric productivity by the optimized enzyme cocktail on 10 g/L cellulose and (c) the comparison of iC2S yields in previously reported data and this study. (d and e) Scanning electron microscopy (SEM) images of resins before (d) and after (e) amylose-coating. (f) The chromatographic column packed with the 5-μm resins coated with the amylose derivatives. (g) HPLC chromatogram of *R*-thalidomide and *S*-thalidomide separated by the column packed with amylose-coated resins.

The five-enzyme cocktail, containing CBP, PPGK, PGM, EcAGP (from *Escherichia*  *coli*) and SS, made amylose at the presence of adenosine triphosphate (ATP) and the yield was enhanced with polyphosphate kinase (PPK) for ATP regeneration (Fig. S3a). The addition of pyrophosphatase (PPA) increased the amylose yield to 40.9% by mitigating pyrophosphate inhibition (Fig. S3b). A further increase in polyphosphate-6 concentration raised the amylose yield to 57.8% (Fig. S3c), suggesting that this seven-enzyme pathway was better than the five-enzyme pathway. The EcAGP-containing enzyme cocktail had the highest yield (64.5%) at 10 mM Mg^2+^ (Fig. S4a) because Mg^2+^ was an activator of several enzymes and an inhibitor of EcAGP (Fig. S4b). Therefore, we attempted to identify Mg^2+^-inert AGPs. We employed a phylogenetically guided enzyme-mining approach and constructed a maximum-likelihood phylogenetic tree comprising over 46 AGP homologs with a particular focus on thermostable candidates (Fig. S4e). *Thermotoga maritima* AGP (TmAGP) was identified due to its highest net negative charge. Computational modeling analysis suggested that Mg^2+^ had the lowest effects on TmAGP (Fig. S4c–S4d and S4f–S4m) because the Mg^2+^-resistant activity of TmAGP was associated with its structure stability, which had nearly constant activities regardless of Mg^2+^ levels (Fig. S4a). When TmAGP was used, the enzyme cocktail had an amylose yield of 72.4% at 40 mM Mg^2+^ (Fig. S5a). Furthermore, the enzyme composition of each enzyme was optimized to increase the amylose yield to 83.1% at 37°C and 90.8% at 50°C (Fig. S5c). These data suggested that through reaction condition optimization from pathway design, enzyme choice and temperature to substrate and Mg^2+^ concentrations, the seven-enzyme cocktail achieved a iC2A yield of 97.8% (Figs S3–S5).

The iC2S pathway was composed of two modules: partial hydrolysis of cellulose and the iC2A enzyme cocktail. The optimal pH of the iC2A enzyme cocktail was 7.0–8.0, while two commercial cellulase samples (Novozymes’ Celluclast 1.5L, Lonct’s neutral cellulase) preferred acidic conditions (Fig. S6a). The Novozyme Celluclast 1.5L had the highest hydrolysis ability at pH 7.0 (Fig. S6c). Furthermore, the affinity adsorption of Celluclast on cellulose enabled removal of most β-glucosidase (BG), resulting in cellobiose as the major hydrolysis product (Fig. S6d). The consolidation of partial cellulose hydrolysis and the iC2A enzyme cocktail converted pretreated cellulose to amylose with a yield of up to 97.1%, whereas the negative control (the iC2A enzyme cocktail without cellulase) did not produce amylose (Fig. S7a). Furthermore, the substitution of polyphosphate-6 with polyphosphate-45 enhanced the amylose yield and increased the iC2S rate (Fig. S7b). The iC2S enzyme cocktail containing both low-BG Celluclast and the iC2A seven-enzyme cocktail enabled conversion of 10 g/L cellulose to amylose in the presence of polyphosphate-45 (Fig. 1b). The highest productivity was up to 4.454 g starch/L/h in the first half an hour and the average productivity was 1.142 g starch/L/h.

This synthetic amylose sample, in comparison with cellulose and potato amylose, was characterized by cross-polarization/magic angle spinning ^13^C-nuclear magnetic resonance (CP/MAS ^13^C-NMR) spectroscopy (Fig. S8a), Fourier transform infrared (FTIR) spectroscopy (Fig. S8b) and iodine dye (Fig. 1a). Clearly, all β-glycosidic bonds of cellulose were converted to α-1,4-glycosidic bonds of starch. Different from plant starch from natural or genetically modified crops, this synthetic amylose does not have branches. By tailoring the reaction conditions (e.g. pathway design, enzyme choice, primer concentration, phosphate concentration, temperature, pH and so on), these *in vitro* multiple-enzyme molecular machines can precisely synthesize amylose with different lengths, whose degree of polymerization varied from 52 to 1419, mainly by adjusting the primer concentration (Fig. S8c). It was noted that synthetic amylose samples had a very narrow polydispersity index (PDI). When PGP was chosen instead of *Thermococcus kodakarensis* α-glucan phosphorylase (TkαGP), the PDI values of most samples were less than 1.10 (Table S6).

These excellent-quality amyloses were tested for chromatographic separation. They were chemically modified and then thinly coated on the surface of UniSil® resins (Fig. 1d). A few chiral prodrugs were separated by an high-performance liquid chromatography (HPLC) column packed with amylose-coated resins, such as thalidomide, trans-stilbene oxide, warfarin, flurbiprofen and so on (Fig. S9). Among these, thalidomide is one of the most notorious drugs, responsible for a tragic global medical disaster of limb malformations [8]. Figure 1g shows that *R*-thalidomide can be separated well from teratogenic *S*-thalidomide. Lenalidomide, a derivative of *R*-thalidomide, is now one of the world’s largest small-molecule drugs. This amylose, as a unique separation material of chiral compounds, could be the first high-end application to reach the market.

Enzymatic rearrangement of the glycosidic linkages of cellulose into starch obeys a ‘First-Principle’ route (neither catabolism nor anabolism) and has intrinsic advantage over CO_2_-based, *de novo* synthetic starch [9]. Plants store a huge amount of solar energy as lignocellulosic biomass (i.e. 90 TW), approximately 700-fold of 2.8-billion tonnes of grains as food/feed (e.g. 0.13 TW) [5]. Here, *iv*BT has a great bioprocessing advantage—the consolidation of cellulose hydrolysis and starch synthesis without a cellular membrane. This study doubled the theoretical yield of iC2S, increased actual yields by a factor of five to eight and volumetric productivity by a factor of nearly four (Table S1). Also, we replaced mesophilic enzyme (e.g. potato α-glucan phosphorylase) with thermophilic starch-synthesis enzymes (Fig. 1a) that can work with commercial cellulase at an elevated temperature of 50°C. As a result, decreased cellulase loading was decreased by a factor of three to four. Compared to other artificial photosynthesis aims in starch synthesis, this biotransformation has obvious advantages in both energy efficiency and volumetric productivity (Table S1).

Agricultural industrialization that transmutes cellulose directly into starch in factories could ignite the most profound technological revolution our food system has ever witnessed [3,5]. We believe that accelerating climate change could trigger the next agricultural revolution, just like the birth of modern agriculture that was due to the onset of the Younger Dryas cool period followed by rapid warming. The incoming accelerating climate changes could lead to a new wave of agricultural revolution [10]. The cultivation of perennial plants has great advantages over cultivation of annual crops because perennial plants, without tillage, are associated with expansive root systems, utilize fertilizers more effectively, decrease pesticide usage and labor, protect against soil corrosion, and tolerate severe weather events (such as drought and flood) [4]. In addition, perennial crops have a longer photosynthetic season, enhance biomass productivity and fix more biological carbon in soil [3,5].

In conclusion, this study demonstrated the nearly complete biotransformation of starch from cellulose, with the highest reported starch synthesis productivity (Table S1). It could open the gate to a new agricultural epoch that partially replaces annual starch-rich grain monocultures with perennial biomass plantations married to industrial biomanufacturing refineries.

## Supplementary Material

nwaf503_Supplemental_File
