# Supplementary material for: Complete biotransformation of cellulose to starch in vitro
Source: Natl Sci Rev. 2025 Nov 14;13(1):nwaf503. doi: 10.1093/nsr/nwaf503 (PMC12796820; doi:10.1093/nsr/nwaf503)
Supplement: nwaf503_Supplemental_File [file nwaf503_supplemental_file.pdf]

Supplementary Information for

Complete Biotransformation of Cellulose to Starch *in vitro*

Jingting Wang,[1,2] Yunjie Li, [1,2] Mingyuan Lu,[3] Qingqing Guo,[2] Yuanyuan Chen,[1] Yuan Li,[1,2] Yanhong Jing,[2] Zhenyu Zhai,[2,4] Ting Shi,[1,2] Yuzhen Zhang,[2] Biwang Jack Jiang,[3] Xiang Sheng,[1,4] Yi-Heng P. Job Zhang[1,2,4]*

[1] State Key Laboratory of Engineering Biology for Low-Carbon Manufacturing, Tianjin Institute of Industrial Biotechnology, Chinese Academy of Sciences, 32 West 7th Avenue, Tianjin Airport Economic Area, Tianjin 300308, China

[2] *In vitro* Synthetic Biology Center, Tianjin Institute of Industrial Biotechnology, Chinese Academy of Sciences, Tianjin 300308, China

[3] Suzhou NanoMicro Technology Co Ltd, 2 Baichuan Street, Suzhou Industrial Park, Suzhou, Jiangsu, China

[4] University of Chinese Academy of Sciences, Beijing 100049, China

* Corresponding Author: Y.-H. Zhang (zhang_xw@tib.cas.cn), ORCID: 0000-0002-4010-2250; Tel: (+86)-022-24828768

Contents of this file

1. Materials and Methods

2. Energy Efficiency Calculation

3. Supplementary Tables

4. Supplementary Figures

5. Supplementary References

1. Materials and Methods

Chemicals and materials

All chemicals were analytical-grade or higher and purchased from Sigma-Aldrich (Shanghai, China), Merda (Beijing, China) or Solarbio (Beijing, China), unless otherwise noted. GeneJet plasmid miniPREP kits for plasmid extraction and prestained protein markers were purchased from Thermo Fisher Scientific (Shanghai, China). T4 DNA ligase and DNA polymerase were purchased from New England Biolabs (Beijing, China). Total Starch Assay kit and glucose assay kit were purchased from Megazyme (Wicklow, Ireland). Celluclast 1.5L and Cellic CTec3 from Novozymes were purchased from Sigma. Cellulase samples of LT4, 740 and 8310 were gifted from Qingdao Vland Biotech Co (Qingdao, Shandong, China). Cellulase Lonct acidic and Lonct neutral cellulase were gifted by Shandong Lonct Enzymes Co (Linyi, Shandong, China). Among cellulase samples, beta-glucosidase (BG) can be selectively removed by the affinity adsorption by using pretreated cellulose as described elsewhere [1]. Pretreated-cellulose was prepared from Sigmacell cellulose Type20 as previously described [2], with a modified phosphoric acid treatment of the cellulose solution at 50°C for 30 min [3]. Polyphosphate-6 with a degree of polymerization (DP) of six and polypoosphate-45 (sodium phosphate glass, S4379, DP of 45) were purchased from Sigma-Aldrich. Natural amylose isolated from potato was purchased from Sigma-Aldrich (A0512).

Strains and media

Escherichia coli TOP10 was used as the host cell for DNA manipulation. *E.coli* BL21 (DE3) or Rosetta 2 (DE3) was used as a host cell for the expression of the recombinant protein. The lysogeny broth (LB) medium was used for *E.coli* culture and recombinant protein expression. The final concentrations of antibiotics for *E.coli* culture were 100 mg/L ampicillin, 50 mg/L kanamycin or 34 mg/L chloramphenicol.

Plasmid construction

The DNA sequences coding for *Tm*AGP and *Tm*SS was amplified from the genomic DNA of *Thermotoga maritima*. The DNA sequences encoding for TkPPA and TkαGP were synthesized from Genewiz (Suzhou, Jiangsu, China). All DNA sequences and vector backbone (pET28a and pET20b) were amplified with primers in **Supplementary Table S3**. The two DNA templates were *in vitro* assembled to DNA multimers by prolonged overlap extension PCR [4]. Five microliters of the PCR product containing 1 μg of DNA multimers were transformed to *E.coli* Top10, yielding plasmids in this study **(Supplementary Table S2)**.

Enzyme expression and purification

The strains *E.coli* BL21 (DE3) or Rosetta2 (DE3) containing the protein expression plasmids **(Supplementary Table S2)** were cultivated in the LB medium supplemented with antibiotics appropriately. When A_{600} was reached 0.8-1.0, IPTG was added to a final concentration of 100 μM and cultivation temperature was decreased to 16°C for ~16 h. After centrifugation, the pellets were washed with the normal saline water once and resuspended in 50 mM

HEPES buffer (pH 7.5) containing 100 mM NaCl. The cells were lysed by ultra-sonication followed by centrifugation, the supernatants containing soluble proteins including the target protein were loaded onto Ni-Sepharose™ resin and unbonded proteins were washed out by 50 mM HEPES (pH 7.5) buffer containing 200 mM NaCl, 20 mM imidazole. The target protein was eluted in 50 mM HEPES (pH 7.5) buffer containing 200 mM NaCl, 250 mM imidazole. The eluant was dialyzed against 50 mM HEPES (pH 7.5) buffer containing 100 mM NaCl. Protein concentrations were determined by Bradford reagent (Sigma, B6916) with bovine serum albumin as a standard. Purified enzymes were stored at -80°C for further applications. The purity of protein samples was examined by 12% SDS-PAGE. The SDS-PAGE was stained by Feto SDS-PAGE staining buffer (Life Technologies, BS-18.001).

Activity assay for enzymes

All the enzyme unit was defined as one μmol production produced from substrate per min. Cellobiose phosphorylase (CBP, EC 2.4.1.20) activity for wild-type CBP from *Clostridium thermocellum* (CtCBP) was based on the production of glucose 1-phosphate (G1P) from cellobiose [5]. Reactions were conducted in 100 mM HEPES (pH 7.2) containing 10 mM cellobiose, 10 mM phosphate and 5 mg/L CtCBP at 50°C for 10 min. The reaction was started by the addition of CtCBP and stopped by boiling 10 min. The production G1P was determined as followed: an aliquot (50 μL) was transferred to a cuvette and then mixed with 150 μL of 100 mM HEPES (pH 7.2), 5 mM $MgCl_2$, 2 mM NAD^+, 2 U/mL TkPGM and 2 U/mL mG6PDH. The absorbency at 340 nm due to NADH formation after 10 min was measured at 50°C. The activity of CtCBP was 8 U/mg at 50°C.

Polyphosphate glucokinase (PPGK, EC 2.7.1.63) activity for mPPGK (engineered PPGK from *Thermobifida fusca*) was determined as described previously [6]. The activity of mPPGK was 295 U/mg at 50°C.

ADP-glucose pyrophosphorylase (AGP, EC 2.7.7.27) activities for engineered AGP from *E.coli* (EcAGP) and wild-type AGP from *Thermotoga maritima* (TmAGP) were determined as described previously [7]. The activity of EcAGP was 19.2 U/mg at 37°C [7] and TmAGP was 11.5 U/mg at 50°C.

Starch synthase (SS, EC 2.4.1.21) activities for SS from *E.coli* (EcSS) and wild-type AGP from *Thermotoga maritima* (TmSS) were determined as described previously [7].

Pyrophosphatase (PPA, EC 3.6.1.1) activities for wild-type PPA from *E.coli* (*Ec*PPA) and *T.kodakarensis* (*Tk*PPA) were determined as described previously [7].

Polyphosphate kinase (PPK, EC 2.7.4.1) activities for wild-type PPK from *Rhodobacter sphaeroides* (*Rs*PPK) and *Thermosynechococcus elongatus* (*Te*PPK) were determined as described previously [8].

Glucose 6-phosphate dehydrogenase (G6PDH, EC 1.1.1.49) activity of mG6PDH (a thermostable highly active G6PDH mutant from *Zymomonas mobilis*) was determined as described previously [9]. The activity of mG6PDH was 847 U/mg at 60°C [9]. Phosphoglucomutase (PGM, EC 5.4.2.2) activity for the wild-type PGM from *Thermococcus kodakarensis* was determined as described previously [10]. The activity of TkPGM was 100 U/mg at 70°C [10].

Cellobiose-to-starch experiments

The reactions of cellobiose-to-starch synthesis were conducted at 37°C in 100 mM HEPES (pH 7.0) containing 3.4 g/L cellobiose, 5 mM phosphate, 5 mM $MgCl_2$, 6.1 g/L polyposhphate-6 (i.e., 60 mM phosphate equivalent), 0.2 mM maltotetraose as the primer, unless otherwise noted. The seven-enzyme cocktail contained 1 U/mL of CBP, 1 U/mL of mPPGK, 1 U/mL of PGM, 1 U/mL of AGP, 1 U/mL SS, 0.2 g/L of EcPPA, and 0.5 g/L of PPK. The C2A reaction catalyzed by the five-enzyme cocktail were performed in 10 mM phosphate buffer (pH 7.0) containing 5 mM or 10 mM ATP with or without added 0.5 g/L EcPPA and 10 mM ADP (**Fig. S3a**). The pyrophosphate effects on the C2A reaction were conducted with or without 0.2 g/L EcPPA or 10 mM pyrophosphate (**Fig. S3b**). The C2A reactions with ATP regeneration catalyzed by the seven-enzyme cocktail were optimized by adjusting polyposhphate-6 concentration from 3.1 to 15.3 g/L (i.e., 30-150 mM phosphate equivalent) (**Fig. S3c**). The magnesium ion effects from 0 to 40 mM $MgCl_2$ on the C2A reactions were conducted by the seven-enzyme cocktail, where it contained 12.2 g/L polyposhphate-6 (120 mM phosphate equivalent) and 1 U/mL EcAGP or TmAGP (**Fig. S4a**). The C2A reactions were performed at 37°C or 50°C with 20 mM $MgCl_2$, 12.2 g/L polyposhphate-6, 1 U/mL of AGP, 1 U/mL of SS, 0.2 g/L EcPPA or TkPPA, and 0.5 g/L PPK. The optimized thermophilic C2S was performed with 2 U/mL TmAGP and 2 U/mL TmSS at 50°C (**Fig. S5**). The C2A reactions were performed in the Universal Buffer 4 (UB4) buffer (composed of 20 mM HEPES, 20 mM MES and 20 mM sodium acetate) from pH 4.0 to 8.5 containing 20 mM $MgCl_2$ and 12.2 g/L polyphosphate-6 (**Fig. S6a**).

Cellulose-to-starch experiments

The cellulose solutions were hydrolyzed at 50°C at 5 g/L pretreated-cellulose and 0.025 g/L cellulase, unless otherwise noted. The optimal pH for various commercial cellulase samples were hydrolyzed in a UB4 buffer with a pH value from 4.0 to 8.5. Typical cellulase hydrolysis reactions were performed in 100 mM HEPES buffer (pH 7.0). The total soluble sugars in the supernatant of the enzymatic cellulose reactions can be measured by using the phenol-sulfuric acid method directly [11] and the composition of soluble sugars (i.e., D-glucose and cellobiose) were by HPLC equipped with Bio-Rad HPX-87H column at 60°C with 5 mM H_2SO_4 as a mobile phase at a flow rate of 0.6 mL/min and a refractive index detector [12].

To remove the BG from commercial cellulase sample, selective adsorption of cellulase components by pretreated-cellulose was conducted in an ice-bath as described elsewhere [1].

The C2S reactions were carried out in 100 mM HEPES (pH 7.0) containing cellulose, BG-free cellulase, 5 mM phosphate, 20 mM $MgCl_2$, 12.2 g/L polyposhphate-6 (120 mM phosphate equivalent), 0.2 mM maltotetraose as the primer at 50°C. The seven-enzyme cocktail contained 1 U/mL CBP, 1 U/mL mPPGK, 1 U/mL PGM, 2 U/mL AGP, 2 U/mL SS, 0.2 g/L PPA and 0.5 g/L PPK, unless otherwise noted. The polyphosphate type effect on the C2S reactions were performed with either 12.2 g/L polyposhphate-6 (120 mM phosphate equivalent, DP=6) or 12.4 g/L polyphoshphate-45 (120 mM phosphate equivalent) (**Fig. S7b**). The C2S reactions were conducted with 10 g/L cellulose, BG-free Celluclast 1.5, 60 mM $MgCl_2$ and 27.9 g/L polyposhphate-45 (i.e., 270 mM phosphate equivalent) (**Fig. 1b**).

Biosynthesis of well-defined synthetic amylose

The synthetic amylose was synthesized in 50-100 mM sodium phosphate buffer (pH 6.5) consisted of 200 mM disaccharide (e.g., cellobiose or sucrose) and 10 mM magnesium chloride at 50°C. The two-enzyme cocktails were either sucrose phosphorylase derived from *Bifidobacterium adolescentis* [13] or cellobiose phosphorylase from *Clostridium thermocellum* [14], and the starch phosphorylase mutant from potato [15] or alpha-glucan phosphorylase from *T. martima* [16]. Either 0.025, 0.05, 0.1, 0.2, 0.5 mM maltotetraose or 7.5 mM maltose as the primer was used to make the synthetic amylose with a different degree of polymerization (DP). After the amylose synthesis reaction was completed, an equal volume of absolute ethanol was added to induce starch precipitation. The amylose slurry was centrifuged at 4,000 rpm for 5 min. After the supernatant was carefully discanted, the precipitates were washed twice with 50% ethanol. The precipitates were redissolved in 50% dimethyl sulfoxide (DMSO) for the following characterization and reactions.

Characterization of synthetic amylose

The synthetic amylose was characterized by iodine dying, Fourier transform infrared spectroscopy (FTIR), nuclear magnetic resonance spectroscopy (NMR) and molecular size distributions. One hundred μL of the iodine solution (0.2% I_2 and 5.2% KI) was added to 1 mL of the starch-containing reaction mixture for staining. The FTIR spectroscopy was conducted using a Thermo Nicolet 6700 ATR/FT-IR spectrometer (Thermo Fisher Scientific, Shanghai, China). Two hundred fifty-six scans at a resolution of 6 cm^{-1} were averaged for each sample. All FTIR spectra were subjected to Saviszky–Golay smoothing. The absorbance of the bands obtained were resolved using Voigt distribution function by PeakFit 4.12 software. The synthetic amylose was precipitated by equal volume of 100% ethanol as described previously and freeze-dried for NMR analysis. ^{13}C-cross polarization (CP) NMR experiments were acquired on a Bruker Avance NEO 400 MHz spectrometer (9.4T) using a 4 mm HX probe with a CP contact time of 2 ms, recycle delay of 1.5 s and MAS rate of 12 kHz.

Gel permeation chromatography (GPC) was utilized to determine the weight-average molecular weight (MW_W), number-average molecular weight (MW_n) and polymer dispersity

index (PDI) of the synthesized amylose. The analysis was performed using a Shimadzu LC-40D liquid chromatography system equipped with a refractive index detector. A Shodex Ohpak SB - 806M HQ column was selected, and the mobile phase was 50% DMSO. Shodex^{TM} standard P-82 (pullulan polysaccharide) was used as the GPC calibration standard.

Preparation of chiral stationary phases and its HPLC

Amylose tris(3,5-dimethylphenylcarbamate) was prepared by the reaction of the synthetic amylose with a large excess of 3,5-dimethylphenyl isocyanate in pyridine at 110 °C for 18 h under nitrogen protection. The resulted phenylcarbamoylated amylose derivative was isolated as a methanol-insoluble fraction and washed once by methanol and the insoluble parts were removed by filtration. The soluble fraction was reprecipitated by methanol. After centrifugation, the pellets were dried at 80 °C for 20 h. Approximately five grams of amylose tris(3,5-dimethylphenylcarbamate) was dissolved in 60 mL of tetrahydrofuran (THF). The solution was coated on 20 grams of Unisil 5-1000A silica gel provided by Suzhou NanoMicro Technology Co (Suzhou, Jiangsu, China). After THF had been removed, the coated microporous silica gel was packed in a stainless-steel tube (250 × 4.6 mm) by a slurry method. The packed chromatography column was tested by HPLC for the separation of numerous chiral compounds.

The analysis of chiral compounds was performed using a Shimadzu LC-2030C plus liquid chromatography system equipped with different detectors. All chiral compounds were prepared at a loading concentration of 1 mg/mL. The HPLC system was operated at a flow rate of 1 mL/min and column temperature of 30°C. The injection volume, mobile phase and detector wavelength are detailed in **Supplementary Table S4**.

Molecular dynamics simulations

The structural models for this study were constructed using the experimentally determined crystal structure of EcAGP (PDB ID: 5L6S) [17] and the predicted structure of TmAGP generated by AlphaFold2 [18]. The ternary complexes of both enzymes were built by manually docking substrates into their active sites, with reference to the substrate-bound structures of homologous enzymes (PDB ID: 1YP3 and 1G23) to determine the precise substrate positioning [19,20]. The protonation state of all titratable residues were assessed by using the APBS server with visual verification [21]. Histidine residues were in neutral and the acidic residues (Glu and Asp) were set as deprotonated. Lysine and arginine residues were in their protonated forms. The AMBER force fields FF19SB and GAFF2 were used for protein and substrates, respectively. To obtain the force field parameters for G1P and ATP, their structures were first optimized at the B3LYP/6-31G* level of theory. Then, the atomic partial charges were obtained using the restrained electrostatic potential (RESP) method at the HF/6-31G* level. For Mg^{2+}, the compromise set of parameters for divalent ions was employed, which are based on the combination of the TIP3P water model with the classical 12-6 Lennard-Jones nonbonded model [22]. All quantum mechanical calculations were performed using Gaussian 16 software [23].

The molecular dynamics (MD) simulations were carried out for the systems of the EcAGP·ATP·G1P and TmAGP·ATP·G1P complex. We considered two concentrations (0 mM and 100 mM) of Mg^{2+} ions for each system. The model of the above-mentioned systems was solvated in a TIP3P [24,25] water box extending 12 Å from the protein boundary. Subsequently, Na^{+} ions were added to the system for neutralization. The constructed model represnts the system under the condition without Mg^{2+} ion. Based on this model, certain numbers of Mg^{2+} cations and Cl^{-} anions were added to build the model for the system with 100 mM Mg^{2+} ions. The number of Mg^{2+} ions in the system was calculated based on the total volume of the water box: 47 Mg^{2+} ions for the EcAGPase system and 50 Mg^{2+} ions for the TmAGPase system. These Mg^{2+} ions were added to the system randomly using the LEaP program, and then Cl^{-} ions were added to neutralize the system.

After the system setup, the resulting systems were subjected to two-step minimizations to remove the unreasonable interatomic contacts. First, the complex was restrained using a force constant of 20 kcal/(mol•$Å^2$), while the position of solvent and all ions in the system were fully optimized (2500 steps for the steepest descent and 5000 steps for conjugate gradient). For the second step of minimization, the employed parameters were the same as the first step, but the simulation was performed without any restraint. Subsequently, the system was slowly heated from 0 to 300 K under the NVT ensemble for 200 ps with a 2 fs time step, with a restraint constant of 500 kcal/(mol•$Å^2$) on the protein-substrates complex to avoid any unreasonable movement.

After that, the density of the system was equilibrated for 50 ps under the NPT ensemble at a constant temperature of 300 K and 1.0 bar pressure. In this procedure, Langevin thermostat [26] with collision frequency of 2 ps^{-1} and isotropic position scaling [27] for pressure control were used. Subsequently, 200 ps equilibration with no restraint was performed under the same parameters. Then, a productive MD simulation of 500 ns under the NPT ensemble was performed with a time step of 2.0 fs. The periodic boundary conditions were employed throughout the simulations. A cutoff distance of 10 Å for nonbonded interactions was used, and the bonds involving hydrogen were constrained by using SHAKE [28] method. Three replicates were performed for all the systems. All the productive MD simulations were conducted using the GPU version of the AMBER 20 [29] software package. All the RMSD and RMSF analyses were performed using the CPPTRAJ program [30]. The clustering was carried out for each system to group the trajectories into 10 clusters on the basis of the RMSD of all residues as the distance metric.

2. Energy Efficiency Calculation

The theoretical cellobiose-to-starch energy efficiency (η) was calculated based on the standard enthalpy change of combustion ($\Delta_c H°$) of compounds and listed in the **Appendix Table**.

Calculation of the theoretical iC2S energy efficiency (η_{iC2S})

The theoretical η_{iC2S} (the cellulose-to-starch transformation) can be calculated based on the following equations in several ways.

Cellulose (beta-1,4-($C_6H_{10}O_5$)$_n$) = Starch (alpha-1,4-$C_6H_{10}O_5$)$_n$)

The above reaction can be abbreviated to cellobiose-to-maltose, which one beta-1,4-glycosidic bond between two glucose units is converted to alpha-1,4-glycosidic bond between two glucose units. **Method 1**. Given the very small energy change of glycosidic bonds (~1 kJ/mol from cellobiose to maltose), the energy conversion efficiency is 5602/5603 = 99.98%. This value suggests that beta-1,4-glycosidic bond energy nearly equals alpha-1,4-glycosidic bond energy. **Method 2**. The energy efficiency of cellulose to starch is -2794/-2795 = 99.96%. **So, the energy conversion efficiency from this cellulose to starch pathway is approximately 100%.**

The previous cellulose-to-starch reaction can be written as below [1,3].

Method 1: $(C_6H_{10}O_5)_n$ + n/2 H_2O= $(C_6H_{10}O_5)_{n/2}$ [1/2 Starch (per $C_6H_{10}O_5$)] + n/2 glucose

η_{C2S1} (-2795) / (2*-2794) = 50.02% (~50%)

Method 2: Cellobiose = Starch (per $C_6H_{10}O_5$) + glucose

η_{C2S1} = (-2795) / (-5603) = 49.88% (~50%)

So, the energy conversion efficiency from the previous cellulose to starch pathway was approximately 100%.

The methanol-to-starch reaction is given below [7,31].

6 CH_4O (*aq*) + 3O_2 (*g*) = Starch (per $C_6H_{10}O_5$) + 7H_2O (*l*)

η = (-2795) / (6*-726) = 64.16%

The acetate-to-starch reaction is given below[32],

3$C_2H_4O_2$ (acetate) = Starch (per $C_6H_{10}O_5$) + H_2O

η = (-2795) / (3*-875) = 106.48%

Appendix Table. Thermodynamic data for organic compounds (All values relate to 298.15 K).

Compound	$\Delta_c H°$ (kJ/mol)	Source
α-D-glucose	-2808	[33]
β-D-fructose	-2810	[33]
H_2O	0	[33]
CO_2	0	[33]
Sucrose	-5645	[33]
Methanol	-726	[33]
Acetate	-875	[33]
Cellobiose	-5603	Calculated [a]
Maltose	-5602	Calculated [b]
Starch (per $C_6H_{10}O_5$)	-2795	Calculated [d]
Cellulose (per $C_6H_{10}O_5$)	-2794	Calculated [d]

a, $\Delta_c H°$ (cellobiose) = -2808*2 +13 = -5603 kJ/mol (maltose + H_2O = 2 glucose)

b, $\Delta_c H°$ (maltose) = -2808*2 +14 = -5602 kJ/mol (cellobiose + H_2O = 2 glucose)

c, $\Delta_c H°$ (starch per glucose) = -2808 +13 = -2795 kJ/mol (maltotriose + H_2O = maltose + glucose)

d, $\Delta_c H°$ (cellobiose per glucose) = -2808 +14 = -2794 kJ/mol (cellotriose + H_2O = cellobiose + glucose)

3. Supplementary Tables

Supplementary Table S1. Comparison of starch synthesized from CO_2 or its derivatives and cellulose.

Substrate	Energy	Catalyst	Energy Efficiency (%)		Yield (g/g)	Productivity (mg starch/L/h)	Temperature (Biocatalyst)	Reference
			Theoretical	Actual				
Methanol	NA	Yeast	64.2% [a]	0.58%	0.59%	0.98	30°C	[31]
Acetate	NA	Yeast	106.5% [b]	10.7%	10%	161	30°C	[32]
Methanol	NA	ivBT	64.2% [a]	50.7%	51.3%	410	30°C	[7]
Cellulose	NA	ivBT + microbe	50.0%	14.4%	14.4%	318	30°C	[3]
Corn stove – cellulose	NA	ivBT + microbe	50.0%	18.4%	18.4%	300	30°C	[1]
Cellulose	NA	ivBT	66.7%	41.2%	41.2%	515	40°C	[34]
Cellulose	NA	ivBT	100.0%	93.3%	93.3%	1,142 (average)<br>4,454(peak)	50°C	This study

[a] $6CH_4O + 3O_2 = C_6H_{10}O_5 + 7H_2O$ (**See Supplementary Energy Efficiency Calculation**)

[b] $3C_2H_4O_2$ (acetate) = $C_6H_{10}O_5 + H_2O$ (**See Supplementary Energy Efficiency Calculation**)

Supplementary Table S2. Information of enzymes used in this study.

Enzyme	Abbreviation	EC number	Source organism	Plasmids	Reference
Cellobiose phosphorylase	CBP	2.4.1.20	*Clostridium thermocellum*	pET21a-*Ct*CBP	[35]
Polyphosphate glucokinase	mPPGK	2.7.1.63	*Thermobifida fusca* (engineered)	pET28a-P_{tac}-ppgk-4-1	[6]
Phosphoglucomutase	PGM	2.7.5.1	*Thermococcus kodakarensis*	pET20b-*Tk*PGM-co	[36]
ADP-glucose pyrophosphorylase	*Ec*AGP	2.7.7.27	*Escherichia coli* (engineered)	pET21b-GlgC-mut	[7]
	*Tm*AGP		*Thermotoga maritima*	pET28a-*Tm*AGP	This study
Starch synthase	*Ec*SS	2.4.1.21	*Escherichia coli*	pET21b-GlgA	[7]
	*Tm*SS		*Thermotoga maritima*	pET28a-*Tm*SS	This study
Pyrophosphatase	*Ec*PPA	3.6.1.1	*Escherichia coli*	pET21b-dipase	[7]
	*Tk*PPA		*Thermococcus kodakarensis*	pET28a-gw-*Tk*PPA	This study
Polyphosphate kinase	*Rs*PPK	2.7.4.1	*Rhodobacter sphaeroides*	pET28a-his-*Rs*PPK-co	[37]
	*Te*PPK		*Thermosynechococcus elongatus*	pET28a-*Te*ppk-wt	[8]
glucose-6-phosphate dehydrogenase	mG6PDH	1.1.1.49	*Zymomonas mobilis* (engineered)	pET28a-P_{tac}-ZmG6PDH-Mut4-1	[9]
α-glucan phosphorylase	*Tk*αGP	2.4.1.1	*Thermococcus kodakarensis*	pET20b-*Tk*αGP-CO-HIS	This study
	PGP		*Solanum tuberosum*	pET20b-CtDoc-LL-PGP_CO-No His	[15]

Supplementary Table S3. Information of primers in this study.

Primer	Sequence
*Tm*AGP-IF	gtttaactttaagaaggagatataccatgggaaataccgttgcgatg
*Tm*AGP-IR	gtgctcgagtgcggccgcaagcttctcctccctgactatcacataatctc
*Tm*SS-IF	gtttaactttaagaaggagatataccatgaaagtggtattcgtttcttac
*Tm*SS-IR	gtgctcgagtgcggccgcaagcttccttccaacctttgcaagggcttttttg
*Tk*PPA-IF	gtttaactttaagaaggagatataccatgaacccgtttcatgaactggaac
*Tk*PPA-IR	gtgctcgagtgcggccgcaagcttttctttttgccaaatttttctttatac
28a-VF	aagcttgcggccgcactcgagcac
28a-VR	catggtatatctccttcttaaagttaaac
*Tk*αGP-IF	ctttaagaaggagatatacatatggtgaatgttagcaatg
*Tk*αGP-IR	ctcagtggtggtggtggtggtgctcgaggtcaagtcccttccacttgac
20b-VF	tcgagcaccaccaccaccaccactgag
20b-VR	catatgtatatctccttcttaaag

Supplementary Table S4. HPLC Conditions for separation of chiral compounds.

Chiral compounds		Mobile Phase Composition	Detection Wavelength (nm)
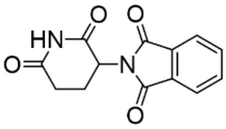	Thalidomide	100% Methanol	220
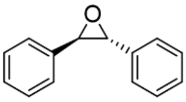	Trans-Stilbene oxide	90% Hexane/10% 2-propanol	254
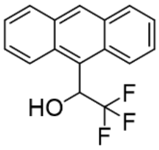	2,2,2-Trifluoro-1-(9-anthryl) ethanol	90% Hexane/10% 2-propanol	254
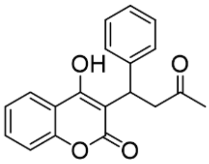	Warfarin	100% Ethanol with 0.1% acetic acid	250
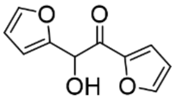	2,2'-Furoin	100% Ethanol	270

Supplementary Table S5. Protein charge analysis of AGP candidates.

Enzyme Uniprot No.	Positive charge	Negative charge	Net charge
B9L1J9	46	50	-4
B7IFV2	35	55	-20
B9K6N9	42	51	-9
**Q9WY82 (TmAGP)**	**42**	**59**	**-17**
B3W9A3	34	40	-6
Q8XP97	47	56	-9
Q9L385	41	46	-5
A3DK82	40	52	-12
B1YK68	41	52	-11
O08326	34	52	-18
Q5SMC1	48	52	-4
O52049	48	48	0
Q6LKA2	40	53	-13
Q2IM42	49	52	-3
Q1DC47	49	49	0
Q8G5Y5	34	50	-16
Q6AF21	42	49	-7
Q6AA20	37	52	-15
Q2JCE9	38	43	-5
C4LHU9	40	56	-16
Q5YQG3	34	50	-16
C1A2N3	38	50	-12
B5XTQ9	46	56	-10
**P0A6V1 (EcAGP)**	**50**	**57**	**-7**
P05415	53	55	-2
Q8ZA77	48	52	-4
Q9CN92	49	56	-7
Q65TI2	47	49	-2
Q13EA6	50	57	-7
Q8U8L5	46	62	-16
Q9RNH7	46	56	-10
Q9ZFN4	44	55	-11
Q47II9	52	57	-5
C5BQ92	46	56	-11
Q21M27	46	56	-10
Q0AA25	46	54	-8
Q6AVT2	60	60	0
P55241	55	60	-5
B8HM61	43	48	-3
Q31QN4	48	52	-4

P30521	49	56	-7
P52417	57	61	-4
P55238	55	60	-5
P55228	55	59	-4
P55232	52	59	-7
P23509	60	61	-1

Supplementary Table S6. Molecular weight characteristics of synthetic amylose.

Degrees of polymerization of synthetic amylose	Weight-average molecular weight (MW_W)	Number-average molecular weight (MW_n)	Polymer dispersity Index (PDI)
52	15054	8350	1.80
202	35062	32795	1.07
404	75782	65508	1.16
652	115421	105636	1.09
871	153660	141104	1.09
1419	289167	229840	1.26

4. Supplementary Figures

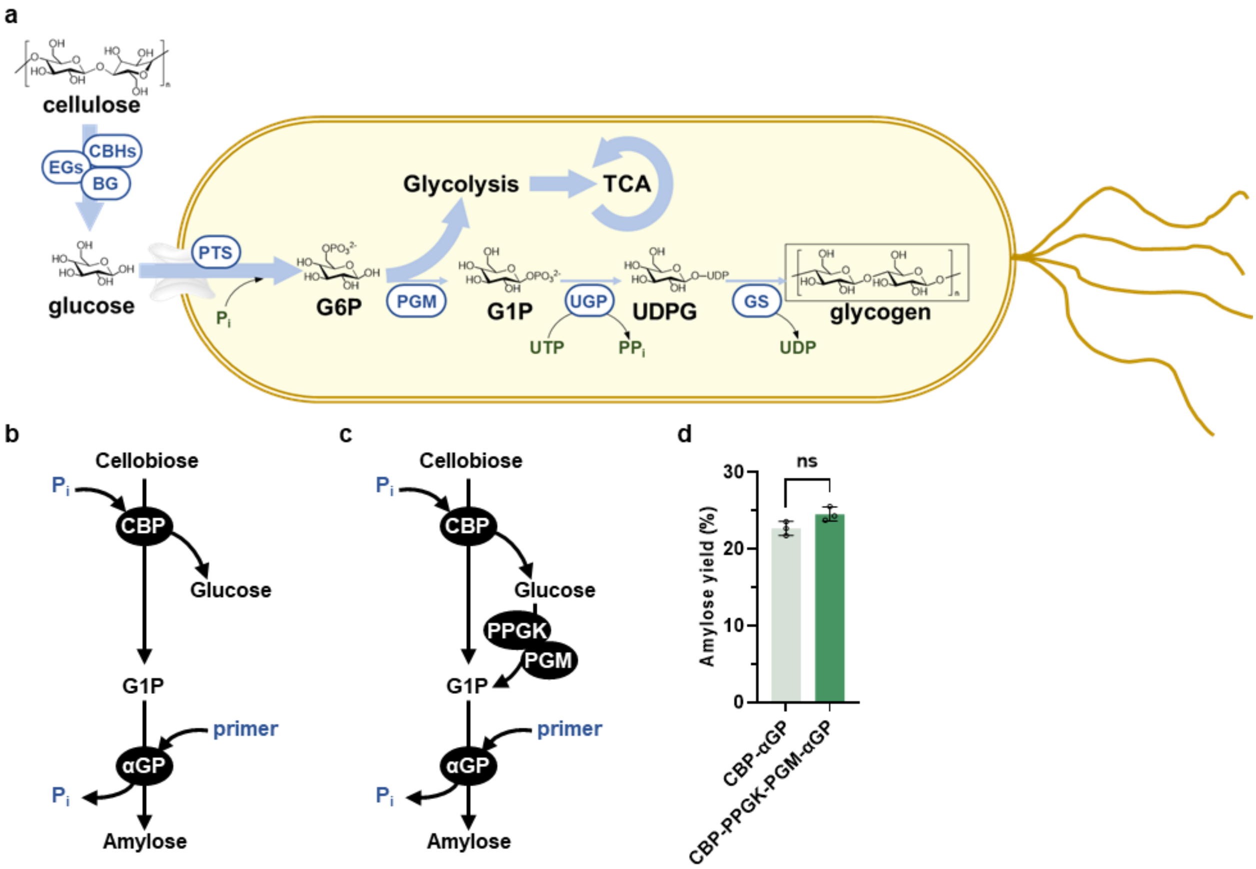

Supplementary Figure S1. Schematic presentation of the *in vitro* cellulose-to-starch (iC2S) biotransformation mediated by a cellulolytic bacterium (a) and enzyme cocktail *in vitro* (b, c). And the amylose yield (c) of these *in vitro* cellulose-to-starch pathways. The data in c represent mean ± SD (standard deviation) obtained in triplicate experiments (n = 3).

EG: endoglucanase, CBH: cellobiose hydrolase, BG: β-glucosidase, PTS: phosphotransferase system, PGM: phosphoglucomutase, UGP: UDP-glucose pyrophosphorylase, GS: glycogen synthase, αGP: α-glucan phosphorylase.

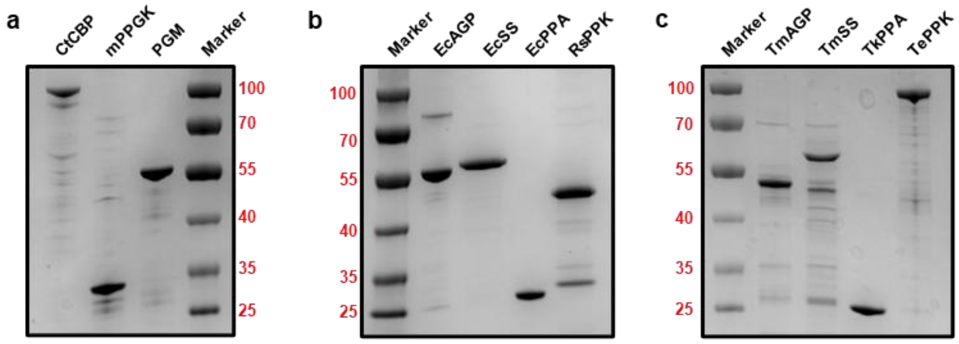

Supplementary Figure S2. SDS-PAGE analysis of all purified enzymes for building C2S pathway.

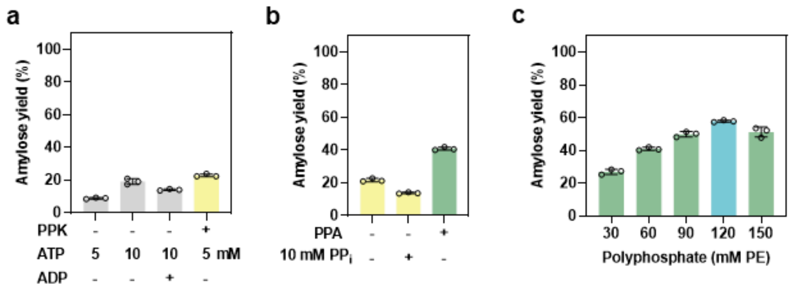

Supplementary Figure S3. The validation and optimization of the iC2A pathway. Effects of the addition of PPK (a), PPA (b) and the concentrations of polyphosphate (c) on the amylose yields. The data represent mean ± SD (standard deviation) obtained in triplicate experiments (n=3).

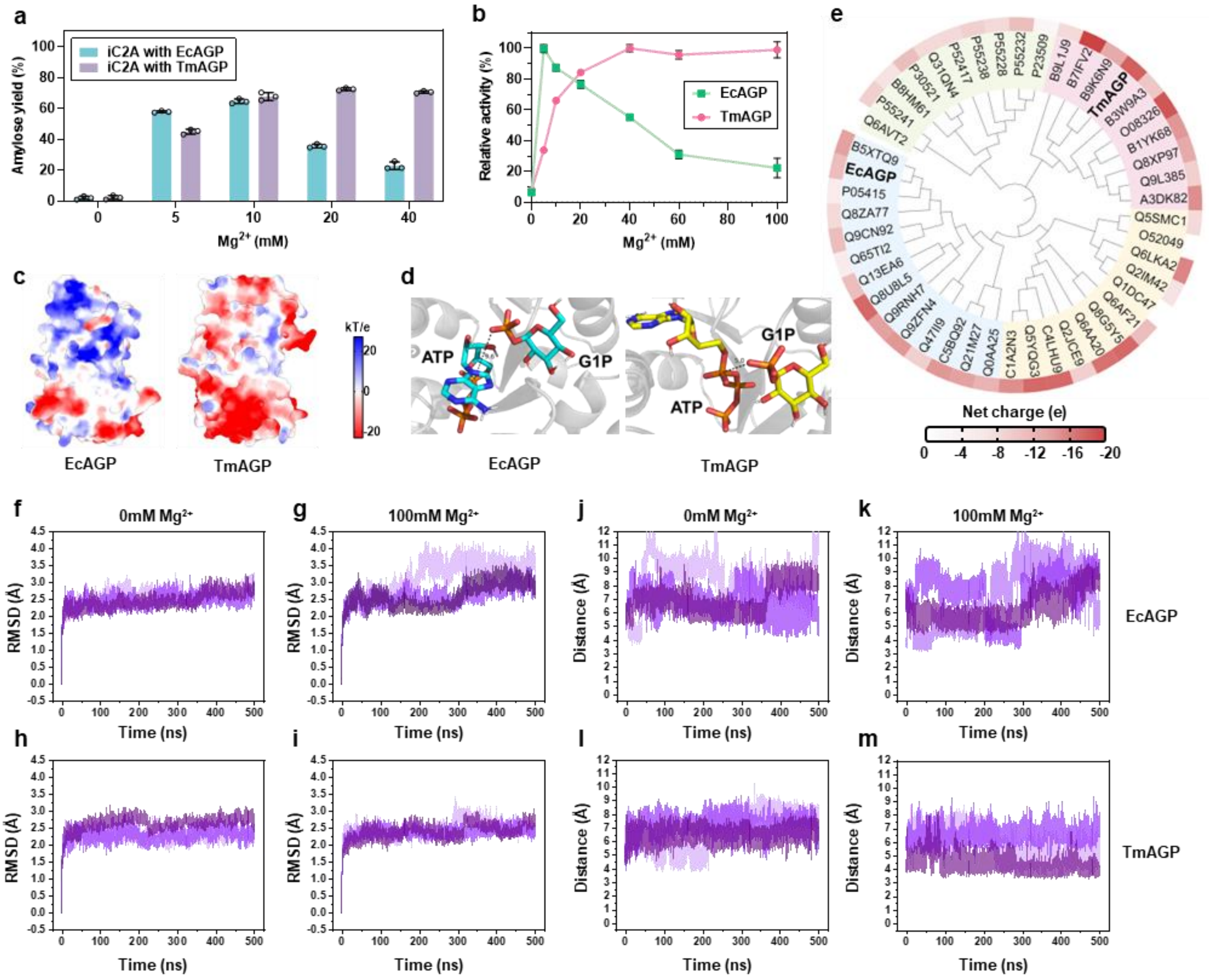

Supplementary Figure S4. The validation and optimization of the iC2A pathway. Effects of the concentrations of magnesium ion on the amylose yields (a) and relative activity (b) of EcAGP or TmAGP. The comparison of charge distributions and represtantive structures from molecular dynamics simulations of AGP from *E. coli* (EcAGP) and a hyperthermophilic bacterium *T. maritima* (TmAGP) (c, d) and a phylogenetic tree with heatmap of net charge mined for Mg^{2+}-insensitive TmAGP (e). The time evolution of the RMSD of EcAGP (f, g) and TmAGP (h, i) during the 500 ns MD simulations with 0 mM Mg^{2+} or 100 mM Mg^{2+}. The fluctuation of the distances (P_{ATP}-O_{G1P}) of EcAGP (j, k) and TmAGP (l, m) during the 500 ns MD simulations with 0 mM Mg^{2+} or 100 mM Mg^{2+}. The three colors correspond to three independent replicates. The data in a and b represent mean ± SD (standard deviation) obtained in triplicate experiments (n=3).

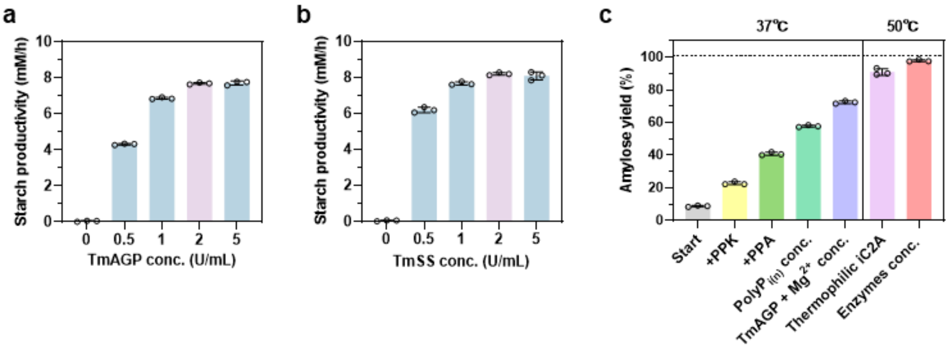

Supplementary Figure S5. Optimization of TmAGP (a) and TmSS (b) concentrations for starch production via iC2A pathway. And the optimization of addition of accessory enzymes, concentrations of Mg^{2+} and polyphosphate, enzyme choice, reaction temperature and enzyme loading on the iC2A yields (c). The data represent mean ± SD (standard deviation) obtained in triplicate experiments (n = 3).

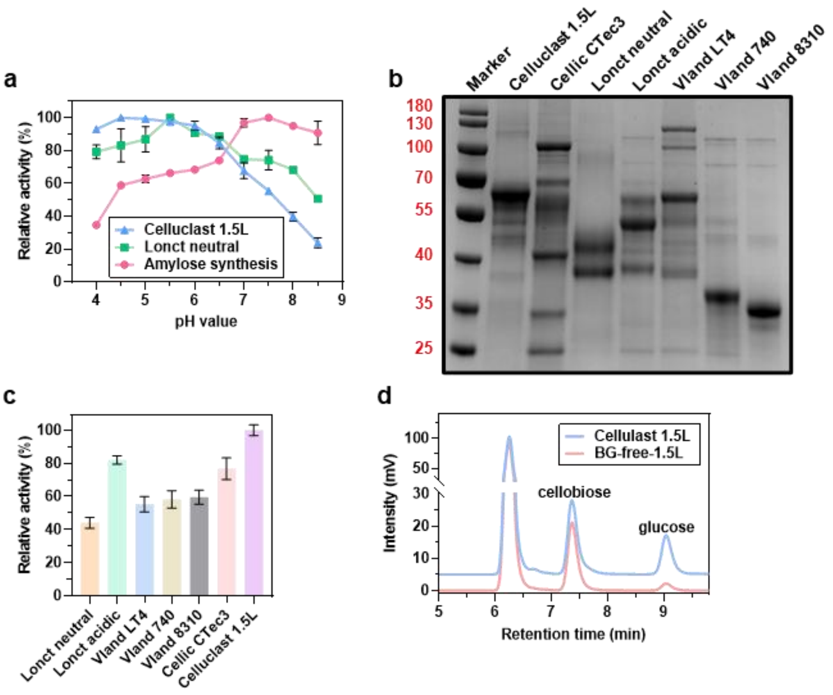

Supplementary Figure S6. The effects of pHs of cellulases and the iC2A cocktail (a). SDS-PAGE analysis of commercial cellulases (b) and these commercial cellulase samples at pH 7.0 (c) on the relative activities. HPLC chromatogram of cellulose hydrolytic products by the commercial Cellulast 1.5L and low-BG Cellulast 1.5L (d). The data represent mean ± SD (standard deviation) obtained in triplicate experiments (n = 3) except b.

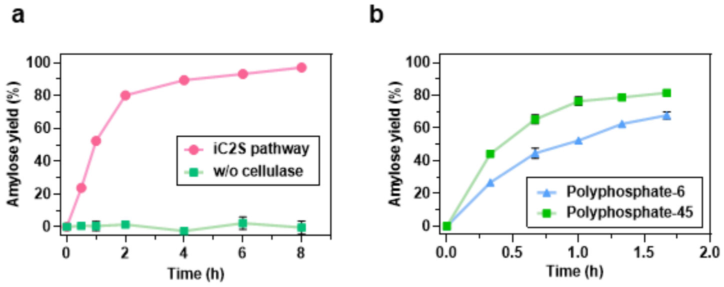

Supplementary Figure S7. The profile of amylose yields catalyzed by the iC2S enzyme cocktail from 10 mM cellulose substrate (glucose equivalent) (a) and effects of the polyphosphate type on amylose yields (b). The data represent mean ± SD (standard deviation) obtained in triplicate experiments (n = 3).

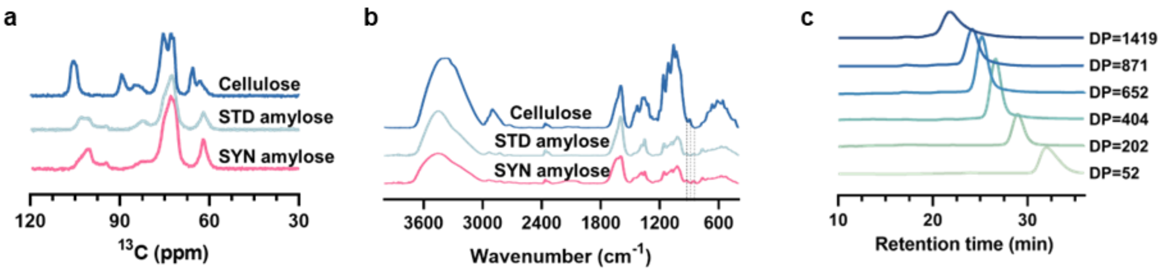

Supplementary Figure S8. The comparison of cellulose, corn starch (STD) and synthetic amylose (SYN) by CP/MAS ^{13}C-NMR (a), and FTIR (b). Size exclusion chromatograms of synthetic amylose with the degree of polymerization (DP) from 52 to 1419 (c).

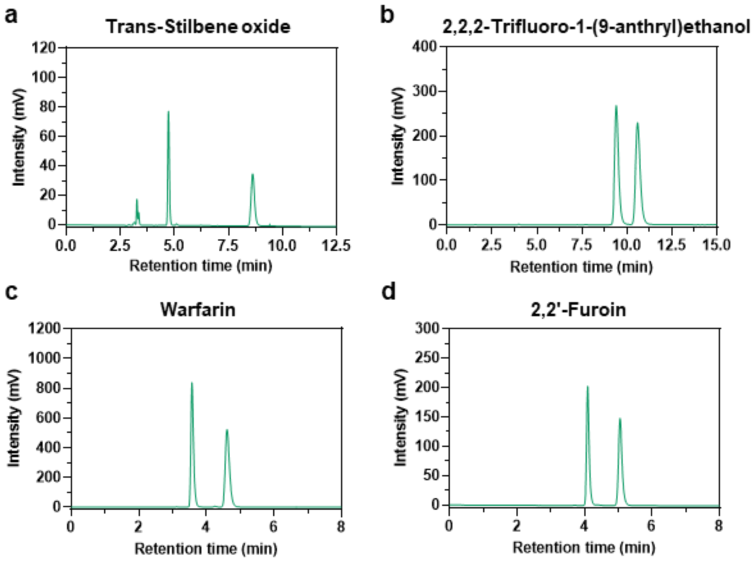

Supplementary Figure S9. HPLC chromatogram of chiral trans-stilbene (a), 2,2,2-trifluoro-1-(9-anthryl) ethanol (b), warfarin (c) and 2,2'-furoin (d) separated by the column packed with amylose-coated resins.

5. References

1. Xu X, Zhang W, You C, et al. Biosynthesis of artificial starch and microbial protein from agricultural residue. *Science Bulletin*. 2023;68(2):214-223. doi:10.1016/j.scib.2023.01.006
2. Zhang YHP, Cui JB, Lynd LR, Kuang LR. A transition from cellulose swelling to cellulose dissolution by o-phosphoric acid: Evidence from enzymatic hydrolysis and supramolecular structure. *Biomacromolecules*. 2006;7(2):644-648. doi:10.1021/bm050799c
3. You C, Chen H, Myung S, et al. Enzymatic transformation of nonfood biomass to starch. *Proceedings of the National Academy of Sciences of the United States of America*. 2013;110(18):7182-7187. doi:10.1073/pnas.1302420110
4. You C, Zhang X-Z, Zhang YHP. Simple Cloning *via* Direct Transformation of PCR Product (DNA Multimer) to *Escherichia coli* and *Bacillus subtilis*. *Applied and Environmental Microbiology*. 2012;78(5):1593-1595. doi:10.1128/aem.07105-11
5. Ye X, Wang Y, Hopkins RC, et al. Spontaneous high-yield production of hydrogen from cellulosic materials and water catalyzed by enzyme cocktails. *Chemsuschem*. 2009;2(2):149-152. doi:10.1002/cssc.200900017
6. Zhou W, Huang R, Zhu Z, Zhang Y-HPJ. Coevolution of both thermostability and activity of polyphosphate glucokinase from *Thermobifida fusca* YX. *Applied and Environmental Microbiology*. 2018;84(16)e01224-18. doi:10.1128/aem.01224-18
7. Cai T, Sun H, Qiao J, et al. Cell-free chemoenzymatic starch synthesis from carbon dioxide. *Science*. 2021;373(6562):1523-1527. doi:10.1126/science.abh4049
8. Sato M, Masuda Y, Kirimura K, Kino K. Thermostable ATP regeneration system using polyphosphate kinase from *Thermosynechococcus elongatus* BP-1 for D-amino acid dipeptide synthesis. *Journal of bioscience and bioengineering*. 2007;103(2):179-184. doi:10.1263/jbb.103.179
9. Huang R, Chen H, Zhou W, Ma C, Zhang YHP. Engineering a thermostable highly active glucose 6-phosphate dehydrogenase and its application to hydrogen production *in vitro*. *Applied Microbiology and Biotechnology*. 2018;102(7):3203-3215. doi:10.1007/s00253-018-8798-7
10. Kim E-J, Kim J-E, Zhang Y-HPJ. Ultra-rapid rates of water splitting for biohydrogen gas production through in vitro artificial enzymatic pathways. *Energy & Environmental Science*. 2018;11(8):2064-2072. doi:10.1039/C8EE00774H
11. Zhang YHP, Lynd LR. Determination of the number-average degree of polymerization of cellodextrins and cellulose with application to enzymatic hydrolysis. *Biomacromolecules*. 2005;6(3):1510-1515. doi:10.1021/bm049235j
12. Zhang YHP, Lynd LR. Cellulose utilization by *Clostridium thermocellum*: Bioenergetics and hydrolysis product assimilation. *Proceedings of the National Academy of Sciences of the United States of America*. 2005;102(20):7321-7325. doi:10.1073/pnas.0408734102
13. Cerdobbel A, De Winter K, Aerts D, et al. Increasing the thermostability of sucrose phosphorylase by a combination of sequence- and structure-based mutagenesis. *Protein engineering, design & selection*. 2011;24(11):829-34. doi:10.1093/protein/gzr042
14. Rollin JA, del Campo JM, Myung S, et al. High-yield hydrogen production from biomass by in vitro metabolic engineering: Mixed sugars coutilization and kinetic modeling. *Proceedings of the National Academy of Sciences of the United States of America*. 2015;112(16):4964-4969. doi:10.1073/pnas.1417719112

15. Yanase M, Takata H, Fujii K, Takaha T, Kuriki T. Cumulative effect of amino acid replacements results in enhanced thermostability of potato type L alpha-glucan phosphorylase. *Applied and environmental microbiology*. 2005;71(9):5433-5439. doi:10.1128/aem.71.9.5433-5439.2005
16. You C, Shi T, Li Y, Han P, Zhou X, Zhang Y-HP. An *in vitro* synthetic biology platform for the industrial biomanufacturing of myo-inositol from starch. *Biotechnology and Bioengineering*. 2017;114(8):1855-1864. doi:10.1002/bit.26314
17. Cifuente Javier O, Comino N, Madariaga-Marcos J, et al. Structural basis of glycogen biosynthesis regulation in bacteria. *Structure*. 2016;24(9):1613-1622. doi:10.1016/j.str.2016.06.023
18. Jumper J, Evans R, Pritzel A, et al. Highly accurate protein structure prediction with AlphaFold. *Nature*. 2021;596(7873):583-589. doi:10.1038/s41586-021-03819-2
19. Jin X, Ballicora MA, Preiss J, Geiger JH. Crystal structure of potato tuber ADP‐glucose pyrophosphorylase. *The EMBO Journal*. 2005;24(4):694-704-704. doi:10.1038/sj.emboj.7600551
20. Blankenfeldt W, Asuncion M, Lam JS, Naismith JH. The structural basis of the catalytic mechanism and regulation of glucose‐1‐phosphate thymidylyltransferase (RmlA). *The EMBO Journal*. 2000;19(24):6652-6663. doi:10.1093/emboj/19.24.6652
21. Jurrus E, Engel D, Star K, et al. Improvements to the APBS biomolecular solvation software suite. *Protein Science*. 2018;27(1):112-128. doi:10.1002/pro.3280
22. Li P, Roberts BP, Chakravorty DK, Merz KM, Jr. Rational design of particle mesh ewald compatible Lennard-Jones parameters for +2 metal cations in explicit solvent. *Journal of Chemical Theory and Computation*. 2013;9(6):2733-2748. doi:10.1021/ct400146w
23. *Gaussian 16 Rev. B.01*. 2016.
24. MacKerell AD, Jr., Bashford D, Bellott M, et al. All-atom empirical potential for molecular modeling and dynamics studies of proteins. *The Journal of Physical Chemistry B*. 1998;102(18):3586-3616. doi:10.1021/jp973084f
25. Jorgensen WL, Chandrasekhar J, Madura JD, Impey RW, Klein ML. Comparison of simple potential functions for simulating liquid water. *The Journal of Chemical Physics*. 1983;79(2):926-935. doi:10.1063/1.445869
26. Izaguirre JA, Catarello DP, Wozniak JM, Skeel RD. Langevin stabilization of molecular dynamics. *The Journal of Chemical Physics*. 2001;114(5):2090-2098. doi:10.1063/1.1332996
27. Berendsen HJC, Postma JPM, van Gunsteren WF, DiNola A, Haak JR. Molecular dynamics with coupling to an external bath. *The Journal of Chemical Physics*. 1984;81(8):3684-3690. doi:10.1063/1.448118
28. Ryckaert J-P, Ciccotti G, Berendsen HJC. Numerical integration of the cartesian equations of motion of a system with constraints: molecular dynamics of n-alkanes. *Journal of Computational Physics*. 1977;23(3):327-341. doi:10.1016/0021-9991(77)90098-5
29. D.A. Case, K. Belfon, I.Y. Ben-Shalom, et al. *AMBER 2020*. 2020.
30. Roe DR, Cheatham TE, 3rd. PTRAJ and CPPTRAJ: Software for processing and analysis of molecular dynamics trajectory data. *Journal of Chemical Theory and Computation*. 2013;9(7):3084-95. doi:10.1021/ct400341p
31. Tang H, Wu L, Guo S, et al. Metabolic engineering of yeast for the production of carbohydrate-derived foods and chemicals from $C_{1–3}$ molecules. *Nature Catalysis*. 2024;7(1):21-34. doi:10.1038/s41929-023-01063-7

32. Shi Z, Xu Z, Rong W, et al. Reprogramming yeast metabolism for customized starch-rich micro-grain through low-carbon microbial manufacturing. *Nature Communications*. 2025;16(1):2784. doi:10.1038/s41467-025-58067-z

33. Peter Atkins JDP, James Keeler. *Atkins' Physical Chemistry*. 11 ed. Oxford University Press; 2017.

34. Zhang Y, Yuan H, Sun C, et al. One-pot biotransformation of cellulose to amylose through multienzyme cascade with nucleoside diphosphate glucose as glycosyl donors. *Carbohydrate Polymers*. 2025;368:124076. doi:10.1016/j.carbpol.2025.124076

35. Ye X, Zhang C, Zhang YHP. Engineering a large protein by combined rational and random approaches: stabilizing the *Clostridium thermocellum* cellobiose phosphorylase. *Molecular Biosystems*. 2012;8(6):1815-1823. doi:10.1039/c2mb05492b

36. Rashid N, Kanai T, Atomi H, Imanaka T. Among multiple phosphomannomutase gene orthologues, only one gene encodes a protein with phosphoglucomutase and phosphomannomutase activities in *Thermococcus kodakaraensis*. *Journal of Bacteriology*. 2004;186(18):6070-6076. doi:10.1128/jb.186.18.6070-6076.2004

37. Liu S, Li Y, Zhu J. Enzymatic production of l-theanine by γ-glutamylmethylamide synthetase coupling with an ATP regeneration system based on polyphosphate kinase. *Process Biochemistry*. 2016;51(10):1458-1463. doi:10.1016/j.procbio.2016.06.006
